# Parietal conditioning enhances motor surround inhibition

**DOI:** 10.1016/j.brs.2019.12.011

**Published:** 2019-12-18

**Authors:** Nivethida Thirugnanasambandam, Giorgio Leodori, Traian Popa, Panagiotis Kassavetis, Alexandra Mandel, Alexander Shaft, Jaron Kee, Sarung Kashyap, Gregg Khodorov, Mark Hallett

**Affiliations:** a Human Motor Control Section, National Institute of Neurological Disorders and Stroke (NINDS), NIH, Bethesda, MD, USA; b IRCCS NEUROMED, Pozzilli, Italy; c The George Washington University, Washington, D.C, USA; d University of Nevada School of Medicine, Reno, NV, USA; e University of New Mexico School of Medicine, Albuquerque, NM, USA

**Keywords:** Transcranial magnetic stimulation (TMS), Dual-site TMS, Motor surround inhibition, Parieto-motor inhibition, Parietal cortex

## Abstract

**Background:**

Motor surround inhibition (mSI) is a phenomenon supportive for executing selective finger movements, wherein synergist muscles are selectively facilitated while surround muscles are inhibited. Previous studies of conditioning inputs to several intracortical and cortico-cortical inhibitory networks did not show an influence on mSI. The inhibitory posterior parietal-motor network, which is crucial for executing fine movements, however, has not been studied.

**Objective/hypothesis:**

To investigate the role of inhibitory posterior parietal-motor network in mSI. We hypothesized that conditioning this inhibitory network would enhance mSI.

**Methods:**

11 healthy adults completed study. mSI was elicited by applying a TMS pulse over the motor cortex coupled with or without a conditioning input to an inhibitory spot in the posterior parietal cortex at 2 or 4 ms interval.

**Results:**

Conditioning input to the posterior parietal cortex increased mSI by ~20%

**Conclusion:**

The inhibitory posterior parietal-motor network appears to contribute to the genesis of mSI.

## Introduction

Motor surround inhibition (mSI) is a neurophysiological phenomenon that selectively facilitates the synergist muscles and inhibits the surrounding muscles to precisely execute a specific motor task [1]. mSI is thought to be mediated by intracortical inhibitory mechanisms since patients with focal hand dystonia (FHD) who exhibited reduced mSI also had less short-latency intracortical inhibition (SICI), a phenomenon mediated by GABAa intracortical networks [2–4]. Concurrent transcranial magnetic stimulation (TMS) and electroencephalography studies also suggest that both mSI and SICI may be mediated by intracortical motor networks [5]. Dual-site TMS has been used to elucidate the role of ipsilateral dorsal and ventral premotor [6,7], and contralateral primary motor cortices in the genesis of mSI, but without success. The anterior part of inferior parietal lobule (aIPL) has inhibitory control over the ipsilateral primary motor cortex (M1) [8] and is also involved in precision grasp [9]. Moreover, FHD patients exhibiting reduced mSI [3,10] also have aberrant parietal-premotor-motor functional connectivity [11,12]. Hence, we speculated that this inhibitory parieto-motor network would influence mSI. In the current study, we aimed to examine the influence of parieto-motor inhibition on mSI and hypothesized that conditioning the aIPL would enhance mSI.

## Methods

We recruited 22 right-handed [13] healthy adults after screening for eligibility to undergo TMS. Any neurological/psychiatric abnormalities, chronic medical/surgical illness, long-term drug intake, metal implants in the body, alcohol abuse and pregnancy were ruled out by a detailed history and physical examination. All subjects gave written informed consent prior to the study. The protocol was approved by the Institutional Review Board at the National Institutes of Health and conformed to the guidelines of the Declaration of Helsinki.

Surface EMG was recorded from the first dorsal interosseous (FDI-synergist) and abductor digiti minimi (ADM-surround) muscles using Ag–AgCl electrodes in a bipolar montage (Nihon-Kohden Neuropack MEB-6300, Japan). The signal was bandpass filtered (20 Hz-2kHz), sampled at 5 kHz using a CED micro1401 laboratory interface and stored for offline analysis using Signal v6.4 (Cambridge Electronic Design Ltd., Cambridge, UK). Dual-site TMS was delivered using 2 custom-made ‘branding iron’ type of figure-of-eight coils (50 mm external diameter) connected to individual Magstim200^2^ stimulators (Magstim, Whitland, UK). MRI-guided neuronavigation (Brainsight, Brainbox Inc., Canada) coupled with an optical tracking system (Polaris Vicra, Northern Digital Inc., Canada) was used to identify and store the stimulation sites for every subject.

Subjects sat on a reclining chair with their right hand resting on a pillow by the side and head fixed in stable position. The motor hotspot for ADM was identified as the one that elicited largest motor-evoked potentials (MEP) consistently. The coil was positioned over the hotspot to deliver a postero-anteriorly directed current in the brain [5]. MEP recruitment curve was plotted as described in previous studies to identify the S50 (intensity that elicits MEP whose amplitude is 50% of the maximum) [5,14]. Resting motor threshold (RMT) was estimated using adaptive threshold hunting procedure [15]. We identified the spot over the aIPL that produced maximum parieto-motor inhibition in individual subjects [8]. To estimate parieto-motor inhibition (PMI), a subthreshold conditioning pulse (90% RMT) over the aIPL followed by a suprathreshold (S50) test pulse over the motor hotspot were delivered at interstimulus interval of 2 or 4 ms. We used the interval that produced maximum inhibition in every subject to condition the aIPL during movement onset.

$$\text{Parieto}-\text{motor inhibition}\left(\text{PMI}\right)=\frac{\text{Mean conditioned MEP amplitude at rest}}{\text{Mean test MEP amplitude at rest}}$$

mSI was estimated using the conventional paradigm [5,16] where subjects performed an auditory-cued brief index finger flexion task with a self-paced delay and suprathreshold pulses were delivered over the ADM hotspot either at rest or at movement onset.

$$\text{mSI}=\frac{\text{Mean MEP amplitude at movement onset}}{\text{Mean MEP amplitude at rest}}$$

In the final block, we coupled parietal conditioning with the mSI paradigm (PM-SI) by administering paired pulses at rest and at movement onset. At least 20 trials were recorded for each condition. PM-SI was calculated as -

$$\text{PM}-\text{SI}=\frac{\text{Mean conditioned MEP amplitude at movement onset}}{\text{Mean test MEP amplitude at rest}}$$

Refer to supplementary information for more details on methodology.

A repeated-measures ANOVA was performed with muscle (2 levels: FDI/ADM) and condition (4 levels: test/mSI/PMI/PM-SI) as independent factors, and normalized peak-to-peak MEP amplitude as dependent variable. Relevant post hoc pairwise comparisons with Bonferroni correction were performed.

## Results

*A priori* power analysis with alpha = 5%, power = 90%, effect size = 25% and standard deviation = 25% (based on pilot data) yielded a sample size of 11 to test our hypothesis effectively. Eleven (mean age in years = 51 ± 11.5(SD); 7 females) of 22 subjects completed the study. The remaining subjects were excluded due to either absent mSI, absent PMI, very high S50, or restricted space to accommodate both coils on the scalp (Supplementary Figure 1). The mean MNI coordinates for the inhibitory aIPL hotspot in our subjects, (x,y,z) = (−56.0 ± 2.5, −53.1 ± 3.0, 50.8 ± 2.5), were consistent with previous studies [8] (Supplementary figure 2).

**Fig. 1.**
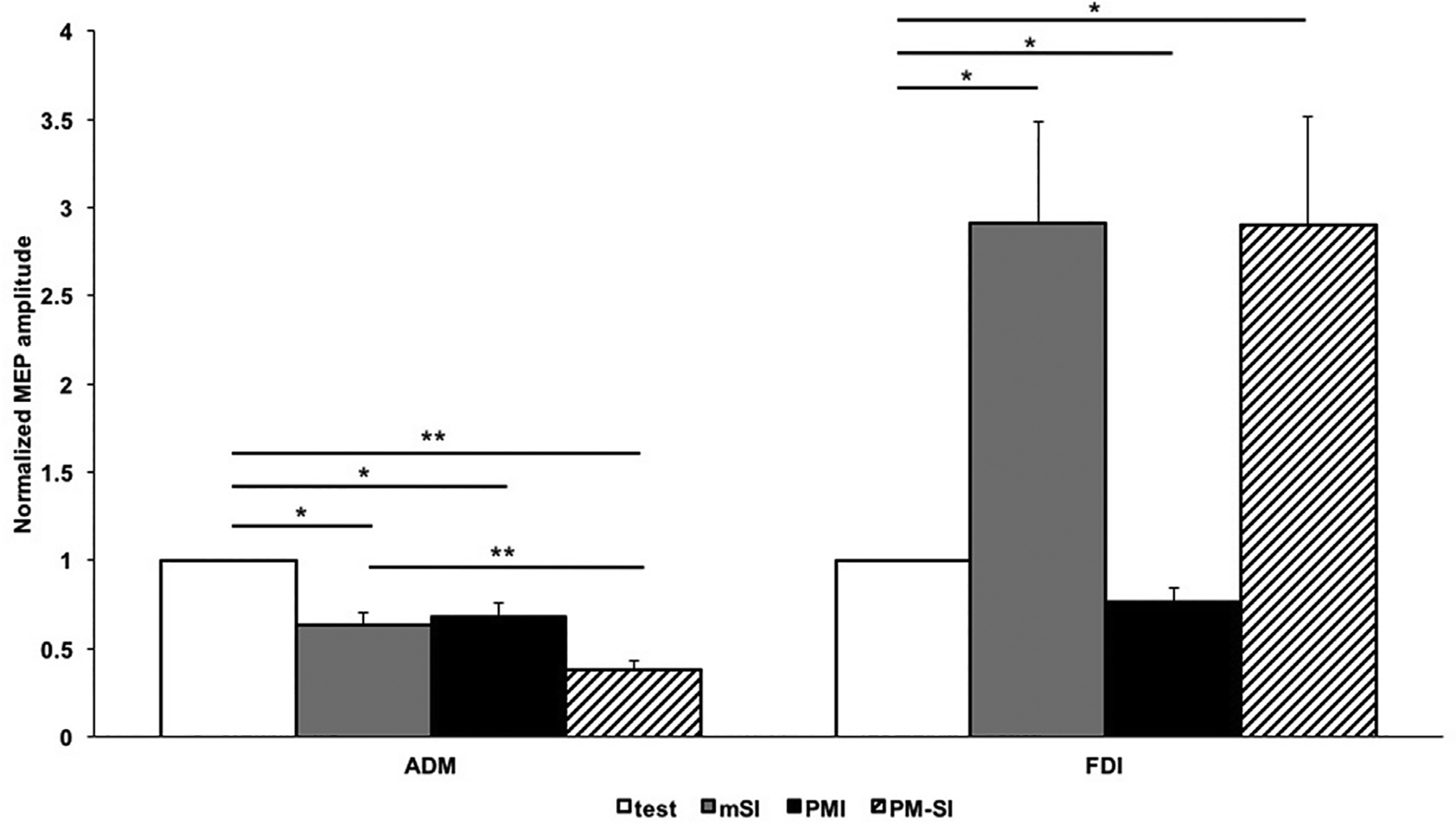
Change in mean MEP amplitude of synergist and surround muscles under different conditions. Shows mean MEP amplitudes normalized to mean test single pulse MEP amplitude for synergist muscle (FDI) and surround muscle (ADM) under different stimulation paradigms: white bar – single test pulse at rest; grey bar – single test pulse at movement onset (motor surround inhibition, mSI); black bar – paired pulse over aIPL and M1 at rest (parieto-motor inhibition, PMI); shaded bar – paired pulse over aIPL and M1 at movement onset (parietal conditioned motor surround inhibition, PM-SI). Error bars indicate standard error of mean. Asterisks indicate p < 0.05. Double asterisks indicate p < 0.001.

ANOVA showed significant main effects of muscle (F(1,10) = 19.508; p = 0.001) and condition (F(3,30) = 9.281; p < 0.001), and their interaction (F(3,30) = 15.090; p < 0.001). Post hoc pairwise comparisons revealed significant PMI for both ADM (p = 0.002) and FDI (p = 0.014). FDI showed significant facilitation at movement onset (p = 0.006). This facilitation remained unaffected by PMI (p = 0.971). Notably, ADM showed significant mSI (p < 0.002). Most importantly, ADM also showed significantly enhanced inhibition for PM-SI (p < 0.001). (See Fig. 1 and Supplementary Table 1). That is, parietal conditioning further enhanced the inhibition of the surround muscle at movement onset.

## Discussion

Our results clearly show that mSI can be significantly enhanced by conditioning the inhibitory parieto-motor network at very short interstimulus intervals thereby suggesting that this is likely to be a monosynaptic connection. Also, since the synergist muscle response remains unaffected, it implies that this inhibitory network selectively influences the surround muscle and, therefore, may contribute to the genesis of mSI. Supporting our results, imaging studies have shown the existence of direct parieto-motor connections independent from the premotor areas embedded in the superior longitudinal fasciculus [9]. In conclusion, the current study indicates that an inhibitory parieto-motor network, possibly monosynaptic, modulates mSI and therefore may be crucial for the generation of mSI. This network may be implicated in encoding precision of fine motor tasks. It is also conceivable that this network may play an important role in the pathophysiology of FHD which should be evaluated in future studies.

## Supplementary Material

Thir et al Brain Stim 2020 Supp data
