# Supplementary material for: Parietal conditioning enhances motor surround inhibition
Source: Brain Stimul. Author manuscript; Available in PMC 2021 Jun 19. (PMC8214400; doi:10.1016/j.brs.2019.12.011)
Supplement: Thir et al Brain Stim 2020 Supp data [file NIHMS1708909-supplement-Thir_et_al_Brain_Stim_2020_Supp_data.docx]

**Supplementary information**

*Subject recruitment*

Our primary hypothesis was that a conditioning input to an inhibitory spot on the posterior parietal cortex would modulate motor surround inhibition (mSI). To test this hypothesis, we compared the degree of mSI elicited by an unconditioned single pulse delivered to the motor hotspot of the surround muscle at movement onset to that elicited by dual-site TMS (conditioning pulse given to the parietal cortex).

For alpha = 5%, power = 90%, estimated effect size = 25% and standard deviation = 25% (obtained from a pilot experiment from our group), the sample size required to test this hypothesis was 11.

Further, to test our hypothesis, it was required that -

1. there were no spatial restrictions to position the 2 coils optimally over the respective stimulation sites,
2. motor surround inhibition (mSI) could be reliably estimated,
3. an inhibitory spot on the parietal cortex (most likely in the anterior part of the inferior parietal lobule (aIPL)) could be identified.

Hence, we screened our subjects prospectively for the above conditions until the required sample size of 11 subjects completed the study. We screened 22 subjects to complete accrual. The complete data from all the 22 subjects are presented in Supplementary table 1.

*Course of the experiment*

Participants were seated on a reclining chair (Brainbox Inc., Canada) with their right arm resting on a table with a pillow to allow complete relaxation. The head and chin were fixed in a stable position. The motor hotspot for ADM (surround muscle) was identified as the one that elicited largest motor-evoked potentials (MEP) consistently. The coil was positioned over the hotspot to deliver a postero-anteriorly directed current in the brain [1]. We recorded the MEP recruitment curve from the ADM as described in previous studies [1, 2] and we identified the stimulation intensity that generated an MEP approximately equal to 50% of the maximum amplitude (S50). We defined the resting motor threshold (RMT) as the minimum stimulation intensity that evoked an MEP of at least 50 µV in 50% of trials using the adaptive threshold hunting procedure [3].

1. *Screening for spatial constraints:*

The 2 TMS coils were positioned in their tentative locations – one over the motor hotspot of the surround muscle and the other over the anatomical aIPL localized on each subject’s MRI. If the coils could be accommodated satisfactorily on their respective locations in a subject, we proceeded further with the experiment. We were able to accommodate both the coils satisfactorily in all subjects and none of the subjects were excluded based on this criteria.

1. *Estimating motor surround inhibition (mSI):*

We used the conventional experimental paradigm for eliciting mSI [1, 4]. A tone was presented once every 10s and the subjects were asked to perform a brief flexion of the right index finger after a self-paced delay on hearing the tone. They were instructed to maintain complete relaxation of the rest of the hand as much as possible. We delivered single TMS pulses over the motor hotspot of the surround muscle (ADM, abductor digiti minimi) randomly either at movement onset, i.e. when the EMG in the synergist muscle (FDI, first dorsal interosseous) reached a threshold of 0.1 mV in amplitude, or at rest, i.e. 5s after the execution of the movement. A total of 30 trials were recorded. The mean MEP amplitude for the two conditions were obtained and the degree of mSI was estimated using the following formula –

$$\text{mSI}\text{ = }\frac{\text{Mean MEP amplitude at movement onset }}{\text{Mean MEP amplitude at rest}}$$

This was repeated twice to reliably assess the mSI in every subject. Only those subjects who consistently had >10% inhibition could proceed with the study. We excluded 10 subjects since they did not show adequate mSI (See Supplementary table 1).

1. *Localizing the inhibitory spot on the parietal cortex:*

From past studies we knew that the aIPL exerted inhibitory control over the primary motor cortex [5]. To estimate the parieto-motor inhibition (PMI), a conditioning pulse was delivered over the anatomically localized aIPL on each subject’s brain MRI followed by a test pulse to the motor hotspot of the surround muscle. The conditioning stimulus (CS) was of subthreshold intensity (90% of RMT) and the test stimulus (TS) intensity was suprathreshold equal to S50. The interstimulus interval (ISI) was either 2 or 4ms. We tested both ISIs because Karabanov and colleagues [6] reported inhibition at both these ISIs. Therefore, this block included 3 conditions (15 trials per condition) occurring in a randomized order: TS only, dual-site CS-TS with 2 ms ISI, and dual-site CS-TS with 4 ms ISI.

Mean MEP amplitude was calculated for each condition and PMI was estimated using the following formula –

$$\text{PMI = }\frac{\text{Mean conditioned MEP amplitude at rest}}{\text{Mean test MEP amplitude at rest}}$$

We chose the ISI that yielded maximum PMI in every subject. If neither ISIs produced at least 10% PMI, the coil delivering the CS was systematically moved in 0.5 cm steps within the anatomical aIPL along a trajectory parallel to the intraparietal sulcus and the block was repeated. Once at least 10% PMI was achieved, the coil position was saved in the neuronavigation system as the ‘inhibitory hotspot' on the parietal cortex. The mean MNI normalized co-ordinates of the inhibitory hotspot were (expressed as mean ± SD): x = -56.0 ± 2.5, y = -53.14 ± 3.0, z = 50.8 ± 2.5, which match well with those reported by Karabanov and colleagues [6]. Supplementary figure 2 shows the inhibitory hotspots in individual subjects. If ≥>10% PMI could not be achieved after repositioning the CS coil 3 times, the subject was excluded from the study. Only one subject out of 12 did not show sufficient PMI and hence was excluded from the study. We did not observe any significant difference between inhibition elicited by 2ms and that by 4ms ISI. A 3-way ANOVA with MEP amplitude as dependent variable and muscle (ADM/FDI), condition (mSI/PMI/PM-SI) and ISI (2ms/4ms) as independent variables revealed neither a significant main effect of ISI nor a significant interaction (condition X ISI or muscle X ISI or muscle X condition X ISI). Of the 11 subjects that completed the study, 6 showed maximum inhibition at 2ms while 4ms ISI elicited more inihibition in 5 subjects Supplementary Table 1.

1. *Estimating the influence of PMI on mSI*

Subjects who showed at least 10% mSI and PMI proceeded to the main experiment block. In this block, we coupled parietal conditioning with the mSI paradigm. That is, we delivered conditioning input to the inhibitory hotspot on the parietal cortex and test pulse to the motor hotspot of the surround muscle at the pre-determined ISI (2 or 4ms) for each subject either at rest or at movement onset in a random order. At least 20 trials were recorded for each condition and the influence of parietal conditioning on mSI was estimated using the following formula -

$$\text{PM-SI = }\frac{\text{Mean conditioned MEP amplitude at movement onset }}{\text{Mean test MEP amplitude at rest}}$$

**Supplementary Figure 1:** is a schematic representation of the experimental protocol.

Subject recruitment

Written informed consent

TMS/EMG:

- Motor hotspot
- MEP recruitment curve
- RMT

Neuronavigation:

Localize aIPL on subject’s MRI

Screening for spatial constraints (n=22)

Estimating motor surround inhibition (mSI) (n=22)

Estimating parieto-motor inhibition (PMI) (n=12)

Estimating influence of parieto-motor

inhibition on motor surround inhibition (PM-SI) (n=11)

Exclude subject

**Supplementary Figure 2:** shows the inhibitory hotspots or the stimulation sites on the parietal cortex for individual subjects.


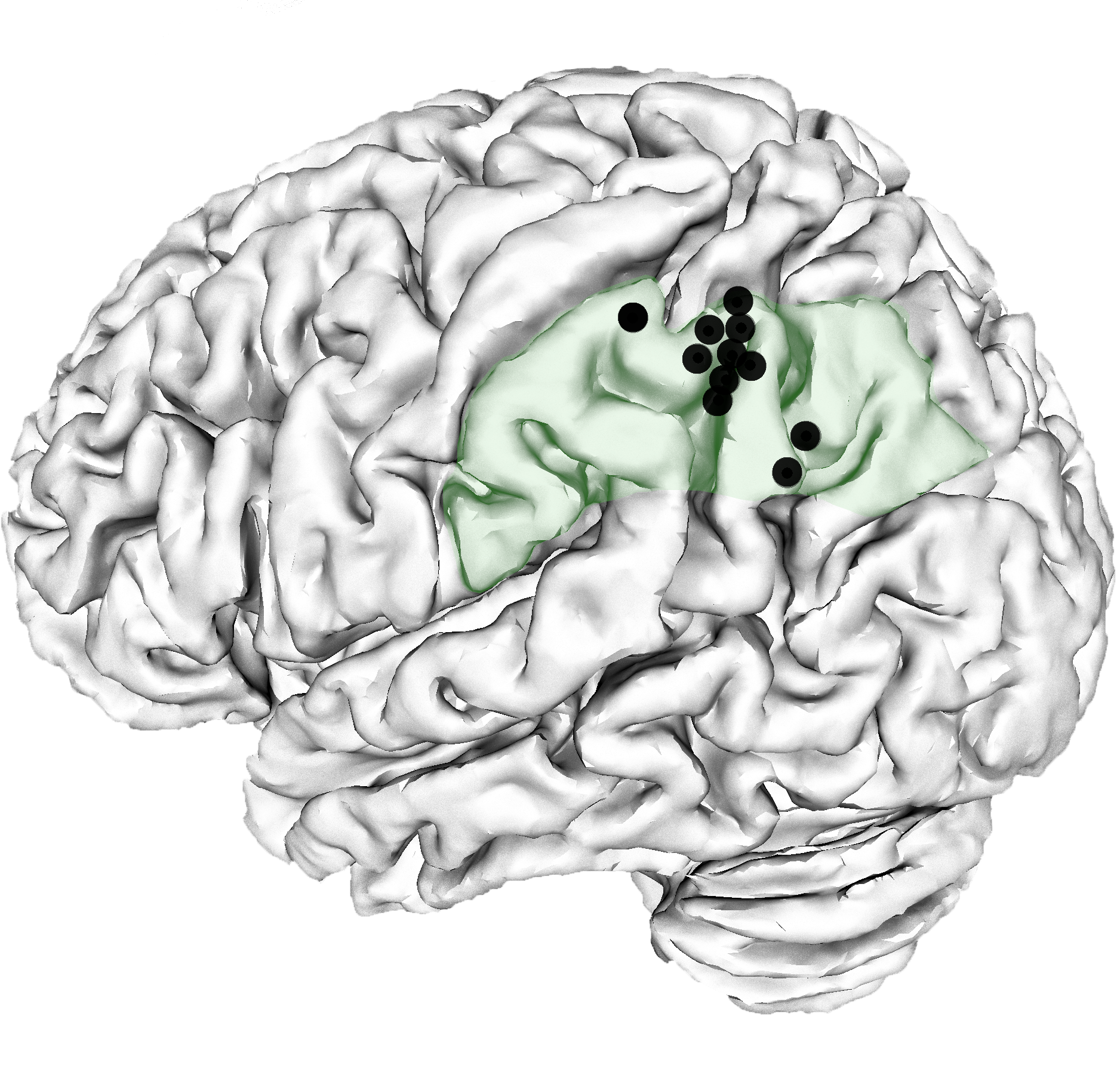


**Supplementary Table 1:** MEP data from all 22 subjects. The 11 subjects who qualified for the final analysis are in bold and italics.

| **Subject** | **SP_MEP (rest)** | **SP_MEP (movt)** | **mSI** | **ISI chosen (ms)** | **PP_MEP (rest)** | **PMI** | **PP_MEP (movt)** | **PM-SI** |
| --- | --- | --- | --- | --- | --- | --- | --- | --- |
| 1 | 0.58 | 0.65 | 1.12 | - | - | - | - | - |
| ***2*** | ***1.36*** | ***0.61*** | ***0.45*** | ***2*** | ***1.24*** | ***0.91*** | ***0.49*** | ***0.36*** |
| ***3*** | ***0.7*** | ***0.34*** | ***0.49*** | ***4*** | ***0.51*** | ***0.73*** | ***0.24*** | ***0.34*** |
| 4 | 1.84 | 3.35 | 1.82 | - | - | - | - | - |
| 5 | 0.18 | 0.26 | 1.44 | - | - | - | - | - |
| 6 | 1.97 | 1.88 | 0.95 | - | - | - | - | - |
| ***7*** | ***4.2*** | ***3.26*** | ***0.78*** | ***2*** | ***1.68*** | ***0.4*** | ***0.57*** | ***0.14*** |
| 8 | 1.93 | 1.85 | 0.96 | - | - | - | - | - |
| 9 | 0.48 | 0.61 | 1.27 | - | - | - | - | - |
| 10 | 1.47 | 0.93 | 0.63 | 2 | 1.52 | 1.03 | - | - |
| 11 | 0.16 | 0.81 | 5.06 | - | - |  | - | - |
| ***12*** | ***2.61*** | ***1.35*** | ***0.52*** | ***4*** | ***1.58*** | ***0.61*** | ***1.72*** | ***0.66*** |
| 13 | 1.76 | 1.91 | 1.09 | - | - | - | - | - |
| ***14*** | ***2.7*** | ***1.61*** | ***0.60*** | ***2*** | ***1.31*** | ***0.49*** | ***0.96*** | ***0.36*** |
| 15 | 0.26 | 0.45 | 1.73 | - | - | - | - | - |
| 16 | 7.8 | 8.68 | 1.11 | - | - | - | - | - |
| ***17*** | ***1.72*** | ***0.35*** | ***0.20*** | ***2*** | ***2.02*** | ***1.18*** | ***0.32*** | ***0.19*** |
| ***18*** | ***2.51*** | ***2.31*** | ***0.92*** | ***4*** | ***1.60*** | ***0.64*** | ***1.61*** | ***0.64*** |
| ***19*** | ***1.08*** | ***0.85*** | ***0.79*** | ***2*** | ***0.40*** | ***0.37*** | ***0.44*** | ***0.41*** |
| ***20*** | ***2.77*** | ***1.91*** | ***0.69*** | ***4*** | ***1.35*** | ***0.49*** | ***0.91*** | ***0.33*** |
| ***21*** | ***1.86*** | ***1.54*** | ***0.83*** | ***4*** | ***1.63*** | ***0.88*** | ***1.20*** | ***0.64*** |
| ***22*** | ***1.14*** | ***0.83*** | ***0.73*** | ***2*** | ***0.91*** | ***0.80*** | ***0.44*** | ***0.38*** |

SP = single pulse; MEP = peak-to-peak amplitude of motor evoked potential in mV; rest = MEP measured at rest; movt = MEP measured at movement initiation; mSI = motor surround inhibition (SP_MEP at movt/SP_MEP at rest); ISI = inter-stimulus interval between conditioning pulse and test pulse in milliseconds; PP = paired pulse where conditioning pulse is delivered to the parietal cortex and test pulse is delivered to the motor hotspot; PMI = parieto-motor inhibition (PP_MEP at rest/SP_MEP at rest); PM-SI = parieto-motor influence on mSI (PP_MEP at movt/SP_MEP at rest)

**References:**

[1] Leodori G, Thirugnanasambandam N, Conn H, Popa T, Berardelli A, Hallett M. Intracortical Inhibition and Surround Inhibition in the Motor Cortex: A TMS-EEG Study. Frontiers in neuroscience 2019;13:612.

[2] Thirugnanasambandam N, Khera R, Wang H, Kukke SN, Hallett M. Distinct interneuronal networks influence excitability of the surround during movement initiation. Journal of neurophysiology 2015;114(2):1102-8.

[3] Awiszus F. TMS and threshold hunting. Supplements to Clinical neurophysiology 2003;56:13-23.

[4] Kassavetis P, Sadnicka A, Saifee TA, Belvisi D, van den Bos M, Parees I, et al. Motor 'surround inhibition' is not correlated with activity in surround muscles. The European journal of neuroscience 2014;40(3):2541-7.

[5] Houdayer E, Beck S, Karabanov A, Poston B, Hallett M. The differential modulation of the ventral premotor-motor interaction during movement initiation is deficient in patients with focal hand dystonia. The European journal of neuroscience 2012;35(3):478-85.

[6] Karabanov AN, Chao CC, Paine R, Hallett M. Mapping different intra-hemispheric parietal-motor networks using twin Coil TMS. Brain stimulation 2013;6(3):384-9.
